# Exercise responses to repeated cycle sprints with continuous and intermittent hypoxic exposure

**DOI:** 10.1002/ejsc.12146

**Published:** 2024-06-14

**Authors:** Siu Nam Li, Prashan Anbalagan, Joel Pang, Mohammed Ihsan, Olivier Girard

**Affiliations:** ^1^ School of Human Sciences (Exercise and Sports Science) The University of Western Australia Perth Western Australia Australia; ^2^ Hong Kong Sports Institute Scientific Conditioning Centre Elite Training Science and Technology Division Hong Kong Hong Kong

**Keywords:** altitude training, environmental stress, hypoxia, internal load, repeated‐sprint ability

## Abstract

We examine the impact of the acute manipulation of oxygen availability during discrete phases (active and passive) of a repeated‐sprint cycling protocol on performance, physiological, and perceptual responses. On separate days, twelve trained males completed four sets of five 5‐s ‘all out’ cycle sprints (25‐s inter‐sprint recovery and 5‐min interset rest) in four randomized conditions: normobaric hypoxia (inspired oxygen fraction of 12.9%) applied continuously (C‐HYP), intermittently during only the sets of sprints (I‐HYP_SPRINT_) or between‐sets recovery periods (I‐HYP_RECOVERY_), or not at all (C‐NOR). Peak and mean power output, peripheral oxygen saturation, heart rate, blood lactate concentration, exercise‐related sensations, and *vastus lateralis* muscle oxygenation using near‐infrared spectroscopy were assessed. Peak and mean power output was ∼4%–5% lower for C‐HYP compared to C‐NOR (*P* ≤ 0.050) and I‐HYP_RECOVERY_ (*P* ≤ 0.027). Peripheral oxygen saturation was lower during C‐HYP and I‐HYP_SPRINT_ compared with C‐NOR and I‐HYP_RECOVERY_ during sets of sprints (∼83–85 *vs*. ∼95%–97%; *P* < 0.001), while lower values were obtained for C‐HYP and I‐HYP_RECOVERY_ than C‐NOR and I‐HYP_SPRINT_ during between‐sets rest period (∼84–85 *vs*. ∼96%; *P* < 0.001). Difficulty in breathing was ∼21% higher for C‐HYP than C‐NOR (*P* = 0.050). Ratings of perceived exertion (*P* = 0.435), limb discomfort (*P* = 0.416), heart rate (*P* = 0.605), blood lactate concentration (*P* = 0.976), and muscle oxygenation‐derived variables (*P* = 0.056 to 0.605) did not differ between conditions. In conclusion, the method of hypoxic exposure application (continuous *vs*. intermittent) affects mechanical performance, while internal demands remained essentially comparable during repeated cycle sprints.

## Highlights

- Continuous hypoxic exposure resulted in reduced repeated sprint performance compared to both normoxia and intermittent hypoxia during recoveries.
- Cardiovascular solicitation and glycolytic contribution showed no significant differences between ambient air and hypoxic conditions regardless of the timing of hypoxic gas administration (entire session, sprints only, or recovery only).
- The method of applying hypoxic exposure significantly affected mechanical performance, while internal physiological demands remained essentially comparable between conditions during repeated cycle sprints.

## 1 INTRODUCTION

Repeated sprint training in hypoxia (RSH) involves the repetition of brief, ‘all out’ exercise bouts interspersed with incomplete recoveries (i.e., exercise‐to‐rest ratio ranging from 1:2 to 1:6), performed in moderate hypoxia [inspired oxygen fraction (FiO_2_) = 13.5% or ∼3000 m simulated altitude] (Faiss et al., 2013; Girard et al., 2017a). Mounting evidence suggests that RSH induces greater improvement in sea‐level mean repeated‐sprint performance [that is, best (fastest sprint time or highest power output) and mean (averaged sprint time or power output recorded/maintained throughout the test) repeated‐sprint ability outcomes] compared with equivalent training undertaken in normoxia for a wide range of combat, endurance, and team‐sport athletes (Brocherie, Girard, et al., 2017; Millet et al., 2019). Potential mechanisms underpinning this improvement include transcription factors that participate in oxygen signaling, enzymes involved in mitochondrial metabolism, enhanced functioning of fast‐twitch muscle fibers through compensatory vasodilation, improved vascular relaxation and increased oxygen supply to the microvasculature as well as a faster rate of phosphocreatine resynthesis (Millet et al., 2019).

Current RSH guidelines involve performing 3–5 sets of 4–7 ‘all out’ efforts lasting 4–15 s with less than 30 s of rest between sprints and 3–5 min of recovery between sets (Brocherie, Girard, et al., 2017). Nonetheless, it should be noted that these recommendations are only a starting point. To achieve optimal implementation, modifying the oxidative–glycolytic balance by manipulating programming variables, such as exercise‐rest ratio of hypoxia severity during the session, can result in specific acute performance and physiological responses. For instance, with a 1:2 exercise‐to‐rest ratio, hypoxia deteriorated performance for sprint durations of up to 10 s (5:10 and 10:20 but not 20:40) during a single repeated sprint exercise session (RSE), while blood lactate concentration increased with sprint duration irrespective of simulated altitude (Raberin, Elmer, et al., 2023). Increasing hypoxia severity likely induces greater stress on the neuromuscular and metabolic regulatory systems leading to larger RSE performance decrements, particularly for FiO_2_ lower than ∼13% (Bowtell et al., 2014; Goods et al., 2014; Townsend et al., 2021). Additionally, modifying the pattern of hypoxic exposure during RSE is a possible strategy that may enhance the activation of regulatory pathways without compromising training quality potentially leading to better outcomes (Brocherie, Girard, et al., 2017).

Recently, Papoti et al. (2023) argued that incorporating hypoxia into exercise sessions may offer added benefits over normoxic conditions but solely if the absolute training intensity is preserved. They propose that inter‐effort recovery hypoxia could harness the advantages of hypoxia exposure without compromising session intensity. Varying the timing of hypoxia provision (i.e., sets of sprints, between‐sets recovery, and both) can arguably provide additional insights regarding oxygen availability on the fractional metabolic contribution during RSE. For instance, recent work investigating the effect or hypoxic (FiO_2_ = 13.6%) or normoxic recovery following ten, 1‐min high‐intensity (120% of the peak aerobic speed or ∼12 km.h^−1^) normoxic running bouts reported an increase in some (higher heart rate and lower SpO_2_) but not all (no differences for blood lactate concentration and ratings of perceived exertion) internal load markers with hypoxic recovery (Dellavechia de Carvalho et al., 2023). However, better understanding of the effects of hypoxia timing could have been obtained by including additional conditions involving sessions conducted entirely in hypoxia or with hypoxic exposure limited to the exercise periods.

An important step toward optimizing RSH prescription is to determine how intermittent hypoxic exposure compares with traditional continuous hypoxia regarding its effect on repeated‐sprint performance as well as physiological and perceptual responses. Oxygen availability during recovery can affect fatigue development during high‐intensity work by influencing the restoration of metabolic homeostasis (i.e., replenishment of phosphocreatine) (Tomlin & Wenger, 2001). It has been reported that muscle reoxygenation capacity is slower during RSE when the recovery periods are undertaken in hypoxia (Billaut & Buchheit, 2013). Assessing changes in muscle oxygenation through near‐infrared spectroscopy (NIRS) may help determine if maintaining normoxia during between‐sets recovery periods of RSE preserves the ability of active musculature to utilize available oxygen during work periods leading to better maintenance of repeated‐sprint performance (Perrey & Ferrari, 2018).

This study examined how exposure to low oxygen conditions during distinct phases (i.e., sets of sprints, between‐sets recovery, and both) of a repeated‐sprint cycling protocol affected acute performance, physiological, and perceptual responses. It was hypothesized that repeated‐sprint performance would be the lowest, and internal load measures would be greater for continuous hypoxia compared to normoxia and to a lower extent intermittent hypoxic exposure.

## 2 METHODS

### 2.1 Participants

Twelve male amateur athletes from various team sports (i.e., water polo, Australian rules football, lacrosse, and soccer) were recruited (age: 25 ± 6 y, body mass: 74.7 ± 12.7 kg, and height: 175.5 ± 8.5 cm). The sample size was determined through a priori power analysis (G*Power 3.1) to detect differences (effect size = 0.71, power of 0.95, and alpha of 0.05) on power output. The effect size was based on previous research examining the effects of normoxic and hypoxic (FiO_2_ = 12.9%) exercise on this variable (Soo et al., 2020), from which a required sample size of 12 participants was determined. The participants lived and trained near the sea level and had not been exposed to altitudes greater than 2000 m in the month prior to testing, were classified as ‘Trained/Developmental’ (Tier 2) using established criteria (McKay et al., 2022). They provided written informed consent prior to the commencement of the study, which was approved by the local Ethics Committee and complied with the Declaration of Helsinki.

### 2.2 Experimental design

This study used a single‐blinded, within‐participant, and cross‐over design. A complete preliminary session was conducted approximately 1 week prior to testing. During this session, participants performed short (<5 s) cycle sprints at increasing intensities while wearing a facemask connected to the portable hypoxic generator (with the hypoxic system turned off) via a ∼1.5‐m long pipe for habituation. The silicone Hans Rudolph facemask was secured over the participants' mouth and nose with a Velcro head strap to ensure ideal support during exercise. The participants were allowed full recovery between sprints and continued until they were comfortable with the sprinting technique (i.e., 3–5 trials). Then, they completed three maximal 5‐s single sprints interspersed with 2‐min of passive recovery followed by one set of the repeated‐sprint exercise test after a 5‐min rest (see *below*). Strong verbal encouragement was given during all maximal efforts.

Participants were instructed to report to the laboratory (well‐ventilated at a constant temperature of ∼22°C and 40% relative humidity) to complete an experimental session on four different occasions 3–7 days apart. Participants performed a repeated‐sprint exercise protocol under each of the following conditions: normobaric hypoxia (inspired oxygen fraction of 12.9%) applied continuously (C‐HYP; hypoxia during the entire session), intermittently during only the sets of sprints (I‐HYP_SPRINT_; hypoxia during sets of sprints only) or between‐sets recovery periods (I‐HYP_RECOVERY_; hypoxia during between‐sets recovery periods only), or not at all (C‐NOR; normoxia during the entire session). All procedures were identical, except for the application of hypoxic exposure during the sprint and/or recovery phases. Participants avoided strenuous exercise, caffeine, and alcohol 24 h prior to all tests, and arrived at the laboratory at the same time of day (±1 h), in a rested and hydrated state, at least 3 h postprandial.

### 2.3 Experimental sessions

Upon arrival, participants were seated for 10 min while being instrumented. Then, they warmed‐up for 10 min while cycling at 100 W (80 rpm) in normoxia. Following 1 min of rest, participants performed five progressive 4‐s submaximal cycling bouts with 30 s of recovery in‐between at a subjective ‘sense of effort’ of 4, 5, 6, 7, and 8 on a modified Borg CR10 ‘sense of effort scale’ (Christian et al., 2014). Following the warm‐up, participants rested passively for 2 min while the facemask was attached. They breathed through the mask for ∼5 s before initiating the repeated‐sprint exercise protocol. Throughout the trial, participants breathed the gas mixture provided by the portable hypoxic generator, which corresponded to their experimental condition.

The repeated‐sprint exercise protocol consisted of four sets of 5 × 5‐s cycle sprints with 25 s and 5 min of passive recovery between sprints and between sets, respectively. All recovery was performed while sitting on the cycle ergometer. A 3‐s countdown was announced prior to each sprint, and strong verbal encouragement was given throughout all sprints. Participants were instructed to perform ‘all‐out’ during the sprints and were routinely given identical instructions (∼5 s before each bout). Heart rate and SpO_2_ were recorded exactly 5 s after each sprint and every 30 s during the 5 min between‐set recovery periods. Ratings of perceived exertion, leg discomfort, and difficulty in breathing were recorded in an invariant order following completion of each set of sprints.

To avoid pacing effects in the repeated‐sprint exercise protocol, participants needed to reach at least 95% of their criterion score (determined from the average of the three reference sprints during the familiarization session). In all testing sessions, all participants met the 95% criteria during the first sprint of the repeated‐sprint exercise protocol, indicating that they did not use anticipatory pacing prior to exercise in any of the trials.

### 2.4 Altitude simulation

Participants wore a facemask with two one‐way valves that restricted inspiration from the top valve and expiration to the bottom valve, which was connected to a portable hypoxic generator (F10 unit, The Altitude Box, Australia). The gas mixture, which was either normoxic (FiO_2_ = 20.9% or ambient air) or hypoxic (FiO_2_ = 12.9% or a simulated altitude of ∼3500 m), was delivered through the top valve. During the C‐HYP and I‐HYP_SPRINT_ trials, participants started inhaling the hypoxic gas ∼5 s before initiating each set of the repeated‐sprint exercise protocol. In the C‐NOR and C‐HYP sessions, participants breathed the normoxia and hypoxia gas mixture, respectively, throughout the test (during sets of sprints and between‐sets recoveries). During I‐HYP_RECOVERY_ and I‐HYP_SPRINT_, hypoxic or normoxic gas mixture were rapidly delivered following the last (fifth) sprint of each set by surreptitiously switching to an FiO_2_ of 12.9% or 20.9%, respectively. The reverse procedure was applied at the end of each 5‐min recovery period, that is, ∼5 s before the commencement of the following set of sprints. This switch during the final ∼5 s of recovery allowed for the hypoxic gas to wash out before the start of the next sprint. The efficacy of the switching procedure between sprint and recovery phases (and vice versa) was verified by monitoring oxygen concentration using a Handi^+^ oxygen monitor (maxtec, Salt Lake City, Utah, US) during preliminary tests. Total hypoxic exposure (i.e., four sets of sprints and four intraset rest periods) during RSE was ∼28 min, ∼8 min, ∼20 min, and 0 min for C‐HYP, I‐HYP_SPRINT_, HYP_RECOVERY_, and C‐NOR, respectively.

### 2.5 Mechanical performance and physiological responses

Repeated‐sprint performance was assessed from measurements of peak power output and mean power output (Cyclemax version 6.3, School of Human Sciences, UWA) for each sprint.

Heart rate was monitored telemetrically with a Polar transmitter–receiver (Bluetooth compatible unit; Polar Electro Oy, Kempele, Finland). Pulse oxygen saturation (SpO_2_) was monitored via fingertip pulse oximetry (Palmsat 2500, Nonin Medical Inc., USA). The heart rate watch (RS400, Polar Electro Oy) and oximeter receiver were attached in a way that prevented participants from seeing any data, in accordance with the blinding procedure.

A capillary blood sample was taken from the fingertip and analyzed for blood lactate concentration using an automated analyzer (Lactate Plus Meter, Nova Biomedical) 2 min after the completion of each of the four sets.

### 2.6 Muscle oxygenation

Muscle oxygenation of the right *vastus lateralis* muscle (∼15 cm above the proximal border of the patella) was monitored noninvasively using a wireless NIRS system weighing 75 g with approximate dimensions (i.e., W × D × H) of 84 × 43 × 17 mm (PortaLite, Artinis Medical Systems, The Netherlands). The system was secured using adhesive tape and elastic bandage to restrict any movement and intrusion of extraneous light. The PortaLite unit consists of three emitter diodes positioned 30, 35, and 40 mm from the detector and emitting infrared light at wavelengths of 760 and 850 nm. All analyses were conducted on data gathered from the 35 mm emitter‐detector distance corresponding to a NIRS signal penetration depth of approximately 17.5 mm. The PortaLite simultaneously uses the modified Beer–Lambert and spatially resolved spectroscopy methods to determine changes in oxygenated (O_2_Hb) and deoxygenated hemoglobin (HHb), expressed in micromolar units (μM) as well as tissue saturation index (TSI, %), which reflects the dynamic balance between oxygen demand and supply within the microcirculation (Ferrari et al., 2004). The system also determines total hemoglobin (tHb = O_2_Hb + HHb), an index representing blood volume capacitance within the area of investigation (Ferrari et al., 2004). All NIRS‐derived data were collected at 10 Hz using a dedicated software (Oxysoft, Artinis Medical Systems) and resampled to 1 Hz for further analyses. Deoxygenation rate (%·s^−1^) was quantified by the slope of TSI decline during sprints, whilst deoxygenation amplitude was determined by the difference between the highest and lowest TSI at the start and at the end of the deoxygenation response, respectively (Cocking et al., 2021). The TSI values at start of each sprint were taken as a proxy indicating the extent of reoxygenation, as reoxygenation rates or amplitude was not determined as leg position and movement were not controlled during the recovery phases. Changes in tHb during sprints were normalized (i.e., ∆ μM) to the minimum value observed following the onset of sprint (Ihsan et al., 2013).

### 2.7 Perceptual responses

Perceptual responses were recorded using the modified Borg CR10 scales (Christian et al., 2014), in an invariant order, immediately after each set. The questions: *“What is your overall perceived exertion?”*, *“How heavy do your legs feel?”* and *“How difficult does it feel to breathe?”* (i.e., with 0 = *“rest”* or *“nothing at all”* to 10 = *“maximal”*) were used for assessing ratings of perceived exertion, leg discomfort, and difficulty in breathing, respectively.

### 2.8 Data and statistical analysis

All data were presented as group mean ± SD. For final analysis, performance, physiological, and perceptual responses were averaged across each set and/or recovery period. Data distribution was assessed via a Shapiro–Wilk test. Two‐way repeated‐measure ANOVAs [condition (C‐HYP, I‐HYP_SPRINT_, I‐HYP_RECOVERY_, and C‐NOR) × time (set 1, 2, 3, and 4)] were used to compare dependent variables followed by *Bonferroni* posthoc analysis procedure adjusted for multiple comparisons. For each ANOVA, partial eta‐squared (*η*_*p*_^2^, with *η*_*p*_^2^ ≥ 0.06 representing a *moderate* effect and *η*_*p*_^2^ ≥ 0.14 a *large* effect) were calculated as measures of effect size. Effect sizes were calculated to determine the magnitude of differences for pairwise comparisons using Cohen's *d*_*z*_ (0.20–0.49 = *small* effect, 0.50–0.79 = *moderate* effect, and ≥0.80 = *large* effect). Statistical analysis was carried out in SPSS (v27; IBM Corp.). Data were considered significant if *P* < 0.05.

## 3 RESULTS

### 3.1 Performance measurements

**FIGURE 1:**
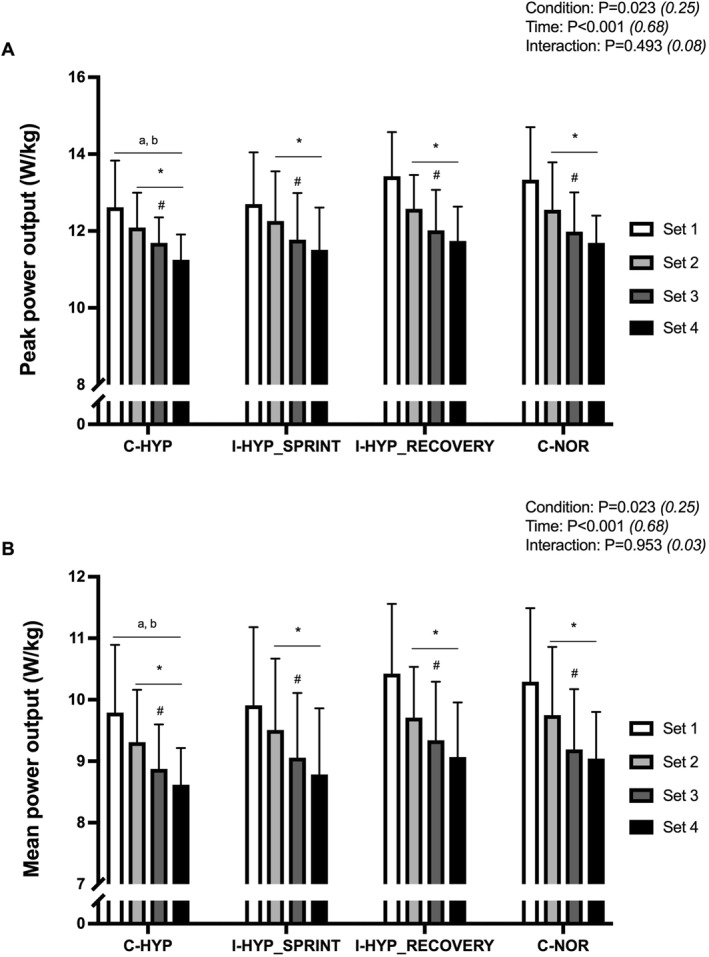
Peak power output (A) and mean power output (B). The repeated‐sprint exercise protocol consisted of four sets five 5‐s cycle sprints with 25‐s of inter‐sprint recovery and 5‐min of interset rest. The exercise was performed with normobaric hypoxia (inspired oxygen fraction of 12.9%) applied continuously (C‐HYP), intermittently during only the sets of sprints (I‐HYP_SPRINT_) or between‐sets recovery periods (I‐HYP_RECOVERY_), or not at all (C‐NOR). Values are means ± SD (*n* = 12). C, T, and I refer to ANOVA main effects of condition, time, and interaction between these two factors with *p*‐value and partial eta‐squared into brackets, respectively. * significantly different from set 1 (*P* < 0.05); ^#^ significantly different from the previous set (*P* < 0.05); ^a^ significantly different from C‐NOR (*P* < 0.05); and ^b^ significantly different from I‐HYP_RECOVERY_ (*P* < 0.05).

Significant main effects were observed for condition (both *P* = 0.023) and time (both *P* < 0.001) in peak and mean power output but not for the interaction between these variables (*P* = 0.493 and *P* = 0.953, respectively) (Figure 1). *Post hoc* analyses indicated significantly lower peak and mean power output averaged values (i.e., pooled for all conditions) during C‐HYP compared to C‐NOR (−4.0 ± 4.4% and −4.5 ± 4.8%; both *P* = 0.050; and *d*_*z*_ ≥ 0.89) and I‐HYP_RECOVERY_ (−4.4 ± 4.4% and −5.3 ± 5.1%; *P* = 0.027 and *P* = 0.023; and *d*_*z*_ ≥ 0.99). Compared to set 1, both peak and mean power output averaged values were significantly lower during sets two (−4.9 ± 2.7% and −5.2 ± 3.4%; both *P* < 0.001; and *d*_*z*_ ≥ 1.50), three (−8.5 ± 5.3% and −9.5 ± 5.8%; both *P* < 0.001; and *d*_*z*_ ≥ 1.54), and four (−10.8 ± 7.2% and −11.6 ± 7.8%; both *P* < 0.001; and *d*_*z*_ ≥ 1.46).

### 3.2 Peripheral oxygen saturation

**FIGURE 2:**
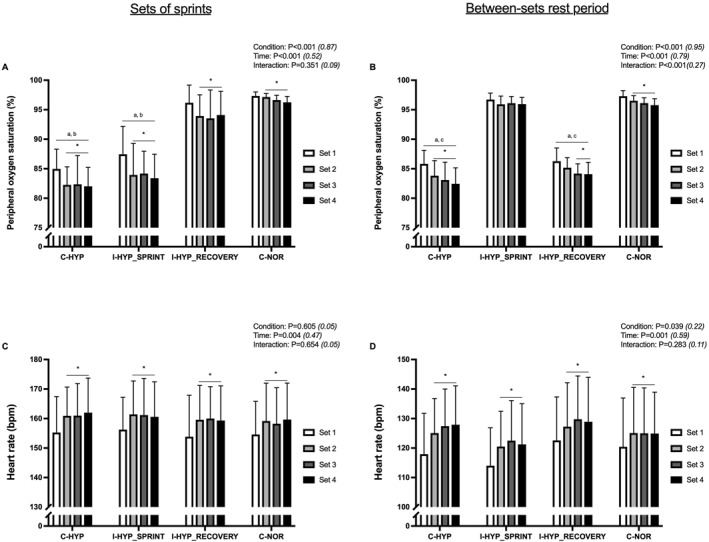
Peripheral oxygen saturation (A and B) and heart rate (C and D) during sets of sprints (left panels) and between‐sets rest period (right panels). The repeated‐sprint exercise protocol consisted of four sets five 5‐s cycle sprints with 25‐s of inter‐sprint recovery and 5‐min of inter‐set rest. The exercise was performed with normobaric hypoxia (inspired oxygen fraction of 12.9%) applied continuously (C‐HYP), intermittently during only the sets of sprints (I‐HYP_SPRINT_) or between‐sets recovery periods (I‐HYP_RECOVERY_), or not at all (C‐NOR). Values are means ± SD (*n* = 12). C, T, and I, respectively refer to ANOVA main effects of condition, time and interaction between these two factors with *p*‐value and partial eta‐squared into brackets. * significantly different from set 1 (*P* < 0.05); ^a^ significantly different from C‐NOR (*P* < 0.05); ^b^ significantly different from I‐HYP_RECOVERY_ (*P* < 0.05); and ^c^ significantly different from I‐HYP_SPRINT_ (*P* < 0.05).

Significant conditions main effects were observed for SpO_2_ during sets of sprints and between‐sets recoveries (Figure 2A,B, respectively). Regarding sets of sprints, significantly lower SpO_2_ values were observed during C‐HYP (82.9 ± 3.2%; *P* < 0.001; and *d*_*z*_ ≥ 2.46) and I‐HYP_SPRINT_ (84.7 ± 3.5% *P* < 0.001; *d*_*z*_ ≥ 2.12, respectively) than C‐NOR (96.8 ± 0.5%) and I‐HYP_RECOVERY_ (94.4 ± 1.0%). Concerning between‐sets recoveries, significantly lower SpO_2_ during C‐HYP (83.8 ± 2.4%; both *P* < 0.001; and *d*_*z*_ ≥ 5.87) and I‐HYP_RECOVERY_ (84.9 ± 1.7%; both *P* < 0.001; and *d*_*z*_ ≥ 5.75) than both C‐NOR (96.4 ± 0.8%) and I‐HYP_SPRINT_ (96.2 ± 1.0%).

### 3.3 Heart rate

A significant main effect of time (*P* = 0.004) was observed for heart rate during sets of sprints (Figure 2C). The averaged heart rate values increased from set 1 to sets 2, 3, and 4 (155 ± 11 *vs*. 160 ± 11, 160 ± 11, and 160 ± 11 bpm, respectively; *P* ≤ 0.05 and *d*_*z*_ ≥ 0.87). For heart rate during between‐sets recoveries, significant main effects were observed for condition (*P* = 0.039) and time (*P* = 0.001) but not for the interaction between these variables (*P* = 0.283) (Figure 2D). The averaged heart rate values increased from set 1 to sets 2, 3, and 4 (119 ± 13 *vs*. 124 ± 12, 126 ± 13, and 126 ± 13 bpm, respectively; *P* ≤ 0.023 and *d*_*z*_ ≥ 1.05).

### 3.4 Blood lactate concentration

**FIGURE 3:**
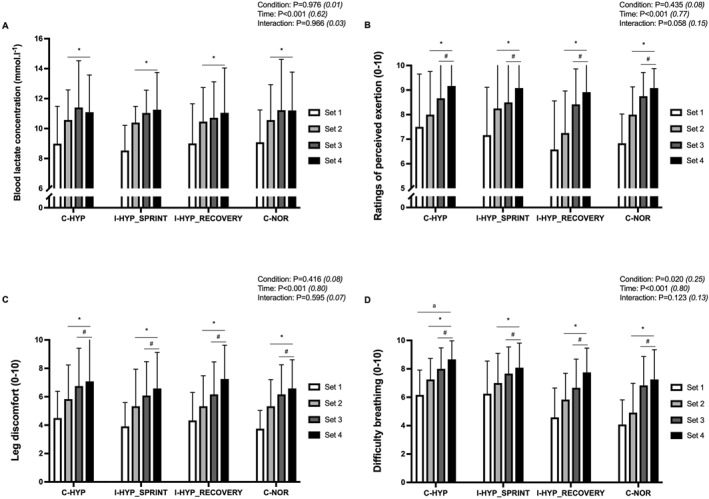
Blood lactate concentration (A), ratings of perceived exertion (B), leg discomfort (C), and difficulty breathing (D). The repeated‐sprint exercise protocol consisted of four sets five 5‐s cycle sprints with 25‐s of inter‐sprint recovery and 5‐min of inter‐set rest. The exercise was performed with normobaric hypoxia (inspired oxygen fraction of 12.9%) applied continuously (C‐HYP), intermittently during only the sets of sprints (I‐HYP_SPRINT_) or between‐sets recovery periods (I‐HYP_RECOVERY_), or not at all (C‐NOR). Values are means ± SD (*n* = 12). C, T, and I, respectively refer to ANOVA main effects of condition, time and interaction between these two factors with *p*‐value and partial eta‐squared into brackets. * significantly different from set 1 (*P* < 0.05); ^#^ significantly different from the previous set (*P* < 0.05); and ^a^ significantly different from C‐NOR (*P* < 0.05).

There was a significant main effect of time (*P* < 0.001) for blood lactate concentration but not for condition (*P* = 0.976) or their interaction (*P* = 0.966) (Figure 3A). Compared to set 1 (8.9 ± 1.4 mmol.L^−1^), blood lactate concentration increased in sets 2, 3, and 4 (10.5 ± 1.4, 11.1 ± 2.0, and 11.2 ± 2.2 mmol.L^−1^; all *P* < 0.001; *d*_*z*_ ≥ 1.35).

### 3.5 Perceptual responses

**FIGURE 4:**
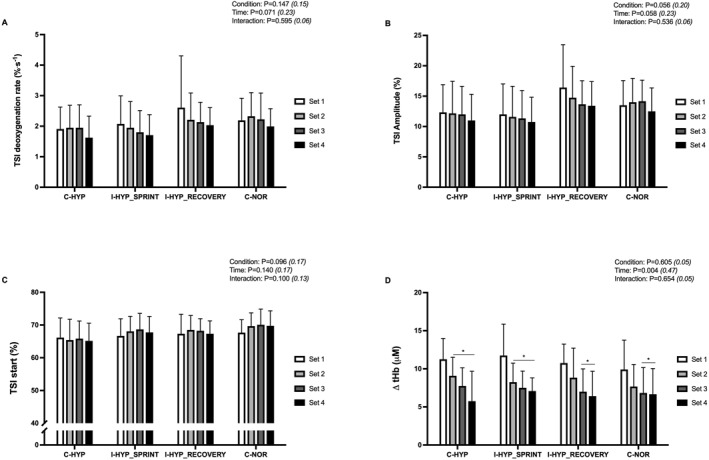
Tissue saturation index (TSI) deoxygenation rate (A), TSI amplitude (B), TSI start (C), and ∆ total hemoglobin (tHb) (D). The repeated‐sprint exercise protocol consisted of four sets five 5‐s cycle sprints with 25‐s of inter‐sprint recovery and 5‐min of inter‐set rest. The exercise was performed with normobaric hypoxia (inspired oxygen fraction of 12.9%) applied continuously (C‐HYP), intermittently during only the sets of sprints (I‐HYP_SPRINT_) or between‐sets recovery periods (I‐HYP_RECOVERY_), or not at all (C‐NOR). Values are means ± SD (*n* = 12). C, T, and I, respectively refer to ANOVA main effects of condition, time and interaction between these two factors with *p*‐value and partial eta‐squared into brackets. * significantly different from set 1 (*P* < 0.05).

The ratings of perceived exertion (*P* = 0.435) and limb discomfort (*P* = 0.416) did not show significant differences between conditions (Figure 4B–C). However, difficulty in breathing was significantly higher for C‐HYP than C‐NOR (+21.4 ± 25.2% *P* = 0.050 and *d*_*z*_ = 0.86) (Figure 4D). Compared to set 1, ratings of perceived exertion (+13.0%–32.4%; all *P* ≤ 0.001 and *d*_*z*_ ≥ 1.72), leg discomfort (+32.1–67.7%; all *P* ≤ 0.001 and *d*_*z*_ ≥ 1.89), and difficulty in breathing (+21.3%–54.8%; all *P* ≤ 0.001 and *d*_*z*_ ≥ 1.43) values were globally elevated in sets 2–4.

### 3.6 Muscle oxygenation

Changes in tHb demonstrated main time effects (Figure 4D). Compared to set 1, significant decreases were evident in sets 2–4 in C‐HYP (*P* = 0.001 to 0.022) and I‐HYP_SPRINT_ (*p* = 0.001 to 0.004) and in sets 3–4 in C‐NOR (*P* = 0.010 to 0.011) and I‐HYP_RECOVERY_ (*P* = 0.007 to 0.009). Time (*P* = 0.071) and condition (*P* = 0.096) effects approached significance for changes in the TSI deoxygenation rate (Figure 4A) and TSI start (Figure 4C), respectively. Condition and time effects (*P* = 0.056 to 0.058) did not reach significance for changes in TSI amplitude (Figure 4B).

## 4 DISCUSSION

Although compelling evidence suggests that repeated‐sprint performance in normobaric hypoxia is attenuated largely due to reduced oxygen availability (Girard, Brocherie, & Millet, 2017), the effect of different hypoxic exposure application methods (continuous *vs*. intermittent) is not fully understood. In this study, changes in external (performance measurements) and internal (physiological responses and exercise‐related sensations) loads during an RSE were examined with continuous (C‐HYP) or intermittent hypoxic exposure [during either sets of sprints (I‐HYP_SPRINT_) or between‐sets recovery periods (I‐HYP_RECOVERY_)], or not at all (C‐NOR). The main findings show decreased mechanical performance in C‐HYP compared with C‐NOR and I‐HYP_RECOVERY_ but not I‐HYP_SPRINT_. Additionally, lower SpO_2_ and higher difficulty in breathing occurred for C‐HYP than C‐NOR (and to a lower extent I‐HYP_RECOVERY_ or I‐HYP_SPRINT_); however, NIRS‐derived muscle oxygenation and blood volume, heart rate, and blood lactate concentration did not differ between conditions. Our results partly confirmed our hypothesis, as we demonstrated that the method of hypoxic exposure application affects mechanical performance while internal demands remained essentially comparable during a multi‐set RSE.

### 4.1 Repeated‐sprint performance

Corroborating with previous RSE studies (Girard, Billaut, et al., 2017), both peak and mean power output values were ∼4%–5% lower for C‐HYP than C‐NOR. This confirms that breathing low oxygen concentrations throughout the session can negatively impact repeated‐sprint performance. However, a novel finding was that mechanical performance in hypoxia was not improved when between‐set recovery was undertaken in normoxia (i.e., I‐HYP_SPRINT_) but was enhanced when sprints were performed in normoxia (i.e., I‐HYP_RECOVERY_). This suggests that reduced oxygen availability mainly affects mechanical performance during the sets of sprints (i.e., active phase) rather than during the between‐sets recovery periods (i.e., passive phase) of a multi‐set RSE. Practically, intermittent hypoxic exposure between sets of sprints may be more advantageous to maintain high absolute mechanical stress during RSE in hypoxia and preserve training quality. In general, our study showed that the impact of hypoxia on performance was relatively modest, despite clear differences in SpO_2_ between normal and low oxygen conditions. This may be due to the short exercise‐to‐rest ratio (1:5) used in our study, with sprint duration being the primary factor explaining repeated‐sprint performance differences between normoxia and moderate hypoxia. In support, increasing sprint duration (5, 10, and 20 s) had a large effect on decrements in power produced during repeated cycling sprints to exhaustion, regardless of simulated altitude (Raberin, Elmer, et al., 2023). In our specific setting, intermittent hypoxia during between‐sets recovery periods may be more effective in maintaining peak and mean power output and thus mechanical strain.

### 4.2 Physiological perturbations

As expected, we observed higher hypoxia‐induced physiological stress as reflected by lower SpO_2_ readings during sprints, recoveries, or both (I‐HYP_SPRINT_, I‐HYP_RECOVERY_, and C‐HYP conditions, respectively) in low oxygen conditions. These SpO_2_ values were comparable to that reported for similar hypoxia severity (i.e., ∼80%–85% at FiO_2_ = ∼13%) (Brocherie, Millet, & Girard, 2017; Goods et al., 2014; Gutknecht et al., 2022). In the RSE literature, the extent to which heart rate responses and/or blood lactate concentrations are exaggerated compared to sea‐level controls is more circumstantial (Girard, Brocherie, & Millet, 2017). For instance, sprint repetition [10 × 6 s all‐out running sprints with 30 s recovery at FiO_2_ = 12–15% (Bowtell et al., 2014) or 3 sets of 9 × 4‐s sprints at FiO_2_ = 12.7% (Goods et al., 2014)] in oxygen‐deprived environments elevates blood lactate concentration. Contrastingly, blood lactate concentration did not differ after a single session of sprint interval training (3 × 30 s sprints) in hypoxia (FiO_2_ = 14.5%) or normoxia, despite causing a greater decrease in muscle glycogen content in normoxia, without interfering with power output (Kaisai et al., 2021). Regardless, we did not observe any differences in these physiological variables between the continuous and intermittent hypoxia application methods in our study. The ‘all‐out’ nature of the exercise protocol may have minimized any potential cardiovascular differences between conditions (Girard, Brocherie, & Millet, 2017). The absence of differences in blood lactate concentration suggests similar contribution of glycolytic pathways. However, it is important to exercise caution when interpreting changes in blood lactate as a measure of intramuscular anaerobic glycolytic rates, as there can be potential discrepancies between blood lactate concentration and muscle lactate production, particularly under hypoxic conditions (Li et al., 2022). Indeed, hypoxia‐induced increase in ventilation (as indicated by higher difficulty breathing scores) can enhance buffer capacity of the extracellular space, which favors lactate efflux from muscles to blood (McLellan et al., 1988). To better understanding the metabolic state of active muscles during hypoxic exposure, measuring blood pH could provide additional insights into the acid‐base state of the extracellular fluid compartment.

#### 4.2.1 Exercise‐related sensations

Both ratings of perceived exertion and leg discomfort did not differ between conditions. However, difficulty in breathing was ∼21% higher for C‐HYP compared to C‐NOR, indicating a heightened sensation of breathlessness in an oxygen‐deprived environment. A previous study that used a similar CR10 Borg scale but a different experimental protocol (10 × 4‐s cycle sprints with 30 s of passive recovery) reported ratings of perceived exertion, limb discomfort, and difficulty breathing values to be 31%–36% higher in hypoxia (FiO_2_ = 13%) compared to normoxia after completing the RSE (Girard, Billaut, et al., 2017). Nonetheless, the comparison of exercise‐related sensations between studies may be limited by differences in experimental protocols such as exercise‐to‐rest ratio, timing of measurement, and mode of hypoxia delivery (i.e., chamber *vs*. mask). Despite this, the current study found that the multi‐set RSE progressively increased the perceptual outcome at a similar rate between conditions. Additionally, maximal scores were not reached during the fourth set, despite the 'all‐out’ nature of the multi‐set RSE involving a total of twenty sprints. Another study that used a similar exercise model and number of sprints (four sets of five, 5‐s running sprints interspersed with 25 s of passive recovery with 5 min of standing rest between sets) found that exercise‐related sensations remained relatively low, with values never exceeding 7 on CR‐10 scales, with or without hypoxic exposure (FiO_2_ = 14.5%) (Brocherie, Millet, & Girard, 2017).

### 4.3 Muscle oxygenation trends

Despite the evident decrease in performance in C‐HYP and the clear impact of hypoxia on SpO_2_ during both the sprint and recovery intervals, changes in muscle oxygenation and blood volume profiles remained relatively similar across conditions. These findings align with recent studies demonstrating comparable muscle oxygenation profiles during RSE in normoxia and normobaric hypoxia (Raberin, Elmer, et al., 2023). Our study expands on these previous findings by showing that introducing hypoxia stimulus during either the sprint or recovery phases of a multi‐set RSE does not significantly influence muscle oxygenation or blood volume. However, it is pertinent to note that similar deoxygenation rates and magnitudes were achieved with lower mechanical power in C‐HYP, indicating a higher oxygen demand relative to supply with C‐HYP and potentially I‐HYP_SPRINT_. Moreover, changes of TSI deoxygenation rates and amplitude displayed lower muscle deoxygenation profiles when sprints were performed in hypoxia (i.e., C‐HYP and I‐HYP_SPRINT_), although statistical significance was not reached. This possibly indicates an increased muscle oxygen demand (relative to supply) when repeated sprints are performed in hypoxia.

## 5 CONCLUSION

In summary, applying hypoxic exposure continuously reduced repeated‐sprint performance compared to both normoxia and hypoxia applied intermittently during recoveries. Additionally, despite greater hypoxic and perceptual strain occurred for C‐HYP than C‐NOR, neither cardiovascular solicitation nor glycolytic contribution differed between ambient air and hypoxic conditions regardless of the timing of the hypoxic gas administration (entire session, sprints only, or recovery only) compared to normal oxygen conditions.

## CONFLICT OF INTEREST STATEMENT

No conflicts of interest to disclose.
